# Glycemic control and blood gas sampling frequency during continuous glucose monitoring in the intensive care unit: A before‐and‐after study

**DOI:** 10.1111/aas.14159

**Published:** 2022-10-28

**Authors:** Johan Mårtensson, Salvatore Cutuli, Fumitaka Yanase, Paolo Ancona, Lisa Toh, Eduardo Osawa, Rinaldo Bellomo

**Affiliations:** ^1^ Department of Physiology and Pharmacology Section of Anaesthesia and Intensive Care, Karolinska Institutet Stockholm Sweden; ^2^ Department of Perioperative Medicine and Intensive Care Karolinska University Hospital Stockholm Sweden; ^3^ Department of Intensive Care Austin Hospital Melbourne Victoria Australia; ^4^ Dipartimento di Scienze dell'Emergenza, Anestesiologiche e della Rianimazione Fondazione Policlinico Universitario Agostino Gemelli IRCCS Rome Italy; ^5^ Australian and New Zealand Intensive Care Research Centre Monash University Melbourne Victoria Australia; ^6^ Department of Critical Care, Melbourne Medical School University of Melbourne Melbourne Victoria Australia; ^7^ Data Analytics Research and Evaluation Center Austin Hospital Melbourne Victoria Australia; ^8^ Intensive Care Unit Royal Melbourne Hospital Melbourne Victoria Australia

**Keywords:** continuous glucose monitoring, critical care, glucose variability, hyperglycemia, type 2 diabetes

## Abstract

**Background:**

Whether subcutaneous continuous glucose monitoring (CGM) can safely replace intermittent arterial blood gas glucose analyses in intensive care unit (ICU) patients remains uncertain. We aimed to compare CGM to blood gas glucose values and assess whether CGM use reduces blood gas sampling frequency and glucose variability in ICU patients with type 2 diabetes managed with liberal glucose control.

**Methods:**

We used the FreeStyle Libre CGM in 15 ICU patients and compared their blood glucose metrics with a pre‐CGM control population of 105 ICU patients with type 2 diabetes. Both groups received insulin to target glucose range of 10–14 mmol/L. We used linear regression analysis adjusted for illness severity to assess the association of CGM use with blood gas sampling frequency and glucose variability. We used mean absolute relative difference (MARD) and Clarke error grid analysis to assess accuracy of matched CGM‐blood glucose values overall, across glucose stata (<10, 10–14, >14 mmol/L), and over time (≤48, 48–96, >96 h).

**Results:**

We analyzed 483 matched glucose values. Overall MARD was 11.5 (95% CI, 10.7–12.3)% with 99% of readings in Clarke zones A and B. MARD was 15.5% for glucose values <10 mmol/L, 11.1% at 10–14 mmol/L, and 11.4% >14 mmol/L. MARD was 13.8% in the first 48 h, 10.9% at 48–96 h, and 8.9% beyond 96 h. CGM use was associated with 30% reduction in blood gas sampling frequency. CGM use was not associated with glucose variability as determined by glycemic lability index or standard deviation of blood glucose.

**Conclusions:**

In our cohort of ICU patients with type 2 diabetes receiving liberal glycemic control, CGM showed acceptable accuracy and was associated with a reduction in blood gas sampling frequency without compromising glucose control. Lowest accuracy was observed at glucose values below 10 mmol/L and during the first 48 h of CGM use.

**Editorial Comment**

In critically ill patients, blood glucose levels are usually monitored with intermittent blood sampling, though means for continuous glucose measurement are becoming available, including through continuous sampling of extravascular fluid. In a cohort of critically ill cases with type 2 diabetes, utility and some possible limits of this sampling method for liberal glucose control treatment were demonstrated.

## 1 INTRODUCTION

High blood glucose variability and hypoglycemia are common among individuals with diabetes treated in the intensive care unit (ICU) and are associated with increased mortality risk.^1^, ^2^, ^3^, ^4^ In contemporary ICUs, insulin therapy is commonly guided by intermittent blood gas glucose analyses, which has a significant impact on staff workload and may counteract timely recognition of hypoglycemia and attenuation of glucose variability. Continuous glucose monitoring (CGM) enables more frequent glucose measurements, thus providing instant feedback on glucose trajectories after interventions and early warnings of impending hypoglycemia. The potential benefits and challenges of introducing CGM systems in the ICU has been highlighted in consensus discussions, raising particular concerns about their accuracy.^5^ However, data from one cluster‐randomized trial demonstrated reduced incidence and severity of hypoglycemia when insulin therapy was guided by an intravenous CGM system.^6^ However, intravascular devices needs frequent calibrations, have been associated with thrombosis,^7^ and may provide potentially unreliable readings in patients receiving intravenous glucose.

Recently, we demonstrated that the subcutaneous FreeStyle Libre™ CGM (Abbott Diabetes Care) has acceptable accuracy when compared with arterial blood glucose in ICU patients with diabetes.^8^ Recent data also suggest that CGM use may reduce the need for point‐of‐care (POC) meter testing and staff workload.^9^, ^10^, ^11^ However, due to its superior accuracy, glucose testing by blood gas analyzers remains the gold standard in critically ill patients and the modality of choice in many ICUs. Yet, no study has so far assessed whether CGM implementation has an effect on the frequency of arterial and venous blood gas testing. Furthermore, no data exist on the impact of CGM on blood glucose variability in the ICU setting.

Therefore, we conducted a before‐and‐after study to assess the association of CGM use with routine blood gas sampling frequency and glucose variability in ICU patients with type 2 diabetes treated according to a liberal glucose control protocol. In addition, we aimed to compare the performance of the FlashLibre CGM to blood gas glucose over different glucose strata and over time.

## 2 METHODS

The study was approved by the ethics committee at Austin Hospital, Melbourne, Australia (approval HREC No. LNR/16/Austin/392) with a waiver for informed consent.

### 2.1 Patients

We included adult (18 years or older) patients with type 2 diabetes admitted to ICU at Austin Hospital, Melbourne, Australia between July 18, 2017 and April 5, 2018 who were expected to remain in ICU for at least 48 h and required insulin to maintain blood glucose within target range (CGM group). We applied the FreeStyle Libre flash glucose sensor (Abbott Diabetes Care) on the patient's upper arm, according to the manufacturers' instructions. After a 1‐h warm‐up, the bedside nurse used the flash reader to scan the sensor on an hourly basis. Additionally, blood gas glucose values were measured at the discretion of the bedside nurse. Before commencing this study, we conducted a pilot accuracy study (including eight patients between August 6 and October 18, 2016) in which bedside nurses were trained in sensor application, startup, and scanning and documentation of CGM data.^8^ According to our ICU protocol for patients with diabetes, introduced in February 2015, intravenous insulin infusion was commenced when glucose exceeded 14 mmol/L and adjusted to maintain a target glucose level between 10 and 14 mmol/L during the entire ICU stay. According to the protocol, hourly blood glucose measurements were recommended to guide insulin therapy (Figure S1). Controls were selected from an existing database including 350 ICU patients with diabetes treated according to our liberal glucose control protocol (target blood glucose 10–14 mmol/L) between February 2015 and April 2016 (No CGM group).^12^ From this database, we included patients with type 2 diabetes who required insulin to reach target glucose range and had at least 10 blood gas glucose values obtained in ICU.

### 2.2 Data collection

We collected all blood gas glucose concentrations obtained during sensor monitoring in CGM patients and during ICU stay in No CGM patients. We measured glucose in arterial or venous blood using the Radiometer ABL825 blood gas analyzer (Radiometer Medical A/S). During the observation period, we calculated the following blood glucose metrics: number of blood gas glucose values per hour; blood gas glucose variability (standard deviation, coefficient of variation, and glycemic lability index^3^); highest blood gas glucose; lowest blood gas glucose; mean blood gas glucose; proportion of blood gas glucose values within target range (10–14 mmol/L); hypoglycemia occurrence (at least one blood gas glucose value below 4 mmol/L). Illness severity was quantified by calculating the Acute Physiology and Chronic Health Evaluation III (APACHE III) score.

### 2.3 CGM performance

We assessed performance (bias and accuracy) overall, in three blood gas glucose strata (<10, 10–14, and >14 mmol/L), and in three strata related to CGM start (0–48, 48–96, and >96 h since sensor start). We estimated bias by calculating the median and mean differences between matched pairs of CGM and blood gas glucose (CGM minus blood gas glucose). Bias was also reported as the median and mean differences expressed in percent of blood gas glucose (median and mean relative difference). We assessed numerical accuracy as the median absolute relative difference (median ARD) and mean absolute relative difference (MARD) expressed in percent of blood gas glucose. We used Clarke error grid analysis to assess clinical accuracy.^13^ Estimates of bias, median ARD and MARD were presented with 95% confidence intervals. For median estimates, we used a binomial method to obtain confidence intervals. Since venous blood glucose may be unreliable in patients receiving intravenous glucose, we also assessed CGM performance compared with arterial and venous blood glucose, respectively, in separate analyses.

### 2.4 Statistical analysis

Continuous data were compared using the Mann–Whitney *U* test. Categorical data were compared using the chi‐square test or the Fisher exact test. We used linear regression analysis to assess the association of CGM use with the number of blood gas glucose measurements per hour and glucose variability (standard deviation, coefficient of variation and glycemic lability index), respectively, before and after adjustment for APACHE III score. Standard deviation, coefficient of variation and glycemic lability index of blood gas glucose were found to be well approximated by log‐normal distributions and were therefore log‐transformed before analysis with results presented as geometric means (95% CI). Due to an imbalance in ICU length of stay between the groups (and that the need for frequent glucose measurements may abate over time), we performed a sensitivity analysis adjusting for this variable. A two‐sided *p* value less than .05 was considered statistically significant. We analyzed data using STATA version 15.1 (Stata Corp.).

## 3 RESULTS

### 3.1 Patients

We included 15 patients with CGM commenced within a median of 28 (IQR 15–75) hours after ICU arrival (CGM group). From our existing database of 350 patients with diabetes managed according to our liberal glucose control protocol we excluded 245 patients (218 not receiving insulin, 16 with less than 10 blood gases, and 11 with type 1 diabetes). Therefore, we included 105 patients with type 2 diabetes who required insulin in the ICU and had at least 10 blood gas glucose values (No CGM group). Patient characteristics are shown in Table 1. As compared to No CGM patients, CGM patients were slightly younger, had lower APACHE III score, and were more likely to be mechanically ventilated on admission, although these differences did not reach statistical significance. Median ICU length of stay was 8.9 (IQR 7.6–14) days in the CGM group and 4.0 (2.3–6.7) days in the No CGM group (*p* < .001).

**TABLE 1:** Patient characteristics

	CGM (*n* = 15)	No CGM (*n* = 105)	*p* Value
Age, years	60 (54–73)	65 (58–75)	.38
Male sex, *n* (%)	10 (66.7)	72 (68.6)	.88
APACHE III score	55 (35–64)	62 (46–79)	.18
HbA1c, %	7.4 (5.9–8.0)	6.9 (6.3–8.0)	.58
Mechanical ventilation on admission, *n* (%)	15 (100)	89 (84.8)	.10
Renal replacement therapy on admission, *n* (%)	1 (6.7)	11 (10.5)	.54
ICU length of stay, days	8.9 (7.6–14)	4.0 (2.3–6.7)	<.001
ICU mortality, *n* (%)	1 (6.7)	18 (17.1)	.46

*Note*: Values are median (IQR) or *n* (%).

Abbreviations: CGM, continuous glucose monitoring; ICU, intensive care unit.

### 3.2 Blood glucose metrics

Blood gas glucose metrics are shown in Table 2. Median number of blood gas glucose values/hour was 0.24 (IQR 0.22–0.30) in the CGM group and 0.34 (IQR 0.29–0.43) in the No CGM group (*p* < .001), suggesting a 30% reduction in sampling frequency. Standard deviation and glycemic labillity index of blood gas glucose were similar whereas coefficient of variation was lower in the CGM group. We observed no significant difference in highest blood gas glucose level. However, lowest and mean blood gas glucose were significantly higher in the CGM group. The proportion of blood gas glucose values within target range was approximately 50% in both groups. No patient in the CGM group and 6.7% of patients in the No CGM group developed hypoglycemia. On linear regression analysis adjusted for APACHE III score, CGM use was associated with lower blood gas sampling frequency (*p* < .001), and lower coefficient of variation of blood gas glucose (*p* = .004). We observed no significant association between CGM use and standard deviation of blood gas glucose (*p* = .25) or glycemic lability index (*p* = .89) (Table 3). Additional adjustment for ICU length of stay did not substantially alter the associations between CGM use and blood gas sampling frequency or glucose variability (Table S1).

**TABLE 2:** Blood gas glucose metrics

Variable	CGM (*n* = 15)	No CGM (*n* = 105)	*p* Value
Number of blood gas glucose values	34 (13–48)	30 (18–49)	.88
Number of blood gas glucose values per hour	0.24 (0.22–0.30)	0.34 (0.29–0.43)	<.001
Standard deviation of blood gas glucose, mmol/L	2.7 (2.0–3.1)	2.8 (2.3–3.5)	.27
Coefficient of variation of blood gas glucose, %	20 (15–23)	23 (19–30)	.006
Glycemic lability index of blood gas glucose, [mmol/L]^2^/h/week	80 (43–139)	89 (59–136)	.68
Highest blood gas glucose level, mmol/L	18 (17–23)	18 (16–19)	.30
Lowest blood gas glucose level, mmol/L	8.8 (7.4–9.8)	6.9 (5.6–7.9)	<.001
Mean blood gas glucose level, mmol/L	14 (13–15)	12 (11–13)	<.001
Proportion of blood gas glucose values within target range^a^, %	50 (31–65)	48 (39–59)	.88
Hypoglycemia^b^, *n* (%)	0	7 (6.7)	.30

*Note*: Values are median (IQR) or *n* (%).

Abbreviation: CGM, continuous glucose monitoring.

^a^ Target blood glucose range was 10–14 mmol/L.

^b^ Blood glucose <4 mmol/L.

**TABLE 3:** Univariable and multivariable linear regression analysis showing the association between CGM use and number of blood gas glucose values and glucose variability (standard deviation, coefficient of variation and glycemic lability index), respectively

Outcome measure	Unadjusted risk estimate (95% CI)	*p* Value	Adjusted risk estimate^a^ (95% CI)	*p* Value
Number of blood gas glucose measurements per hour	−0.11 (−0.17 to −0.06)	<.001	−0.11 (−0.17 to −0.06)	<.001
Standard deviation of blood gas glucose, mmol/L	0.87 (0.72 to 1.05)	.15	0.90 (0.75 to 1.08)	.25
Coefficient of variation of blood gas glucose, %	0.77 (0.65 to 0.90)	.002	0.78 (0.66 to 0.92)	.004
Glycemic lability index of blood gas glucose, [mmol/L]^2^/h/week	0.95 (0.60 to 1.50)	.82	0.97 (0.61 to 1.54)	.89

Abbreviation: CGM, continuous glucose monitoring.

^a^ Adjusted for APACHE III score.

### 3.3 CGM performance

**FIGURE 1:**
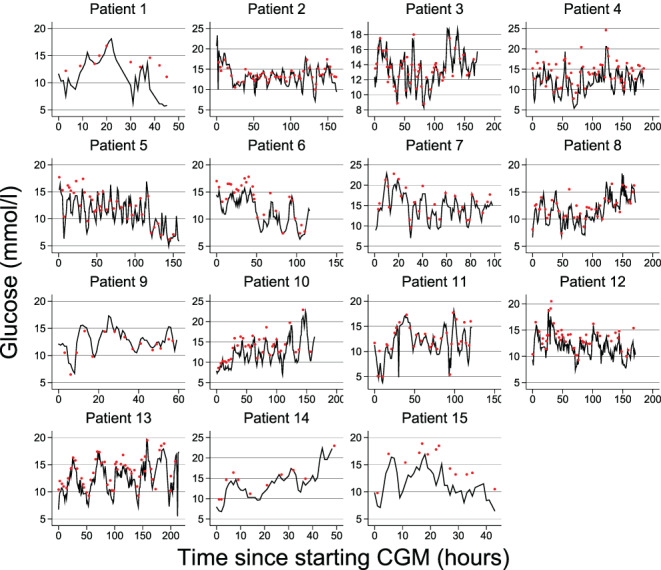
Continuous glucose monitoring values (solid lines) and corresponding blood gas glucose concentrations (red circles) in 15 ICU patients with type 2 diabetes exposed to liberal glucose control

**FIGURE 2:**
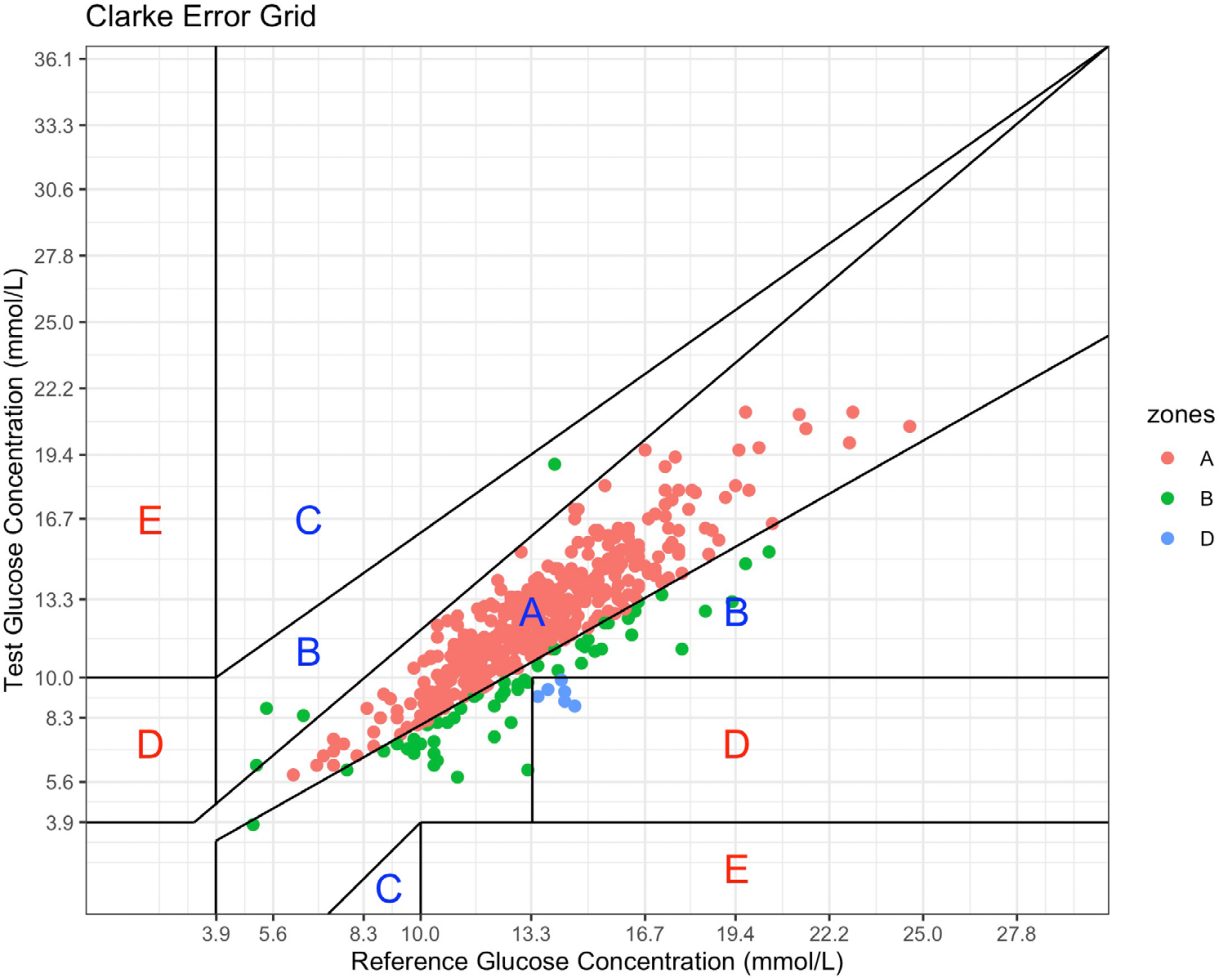
Clarke error grid analysis showing the correlation between continuous glucose monitoring and blood glucose values in 15 critically ill patients with type 2 diabetes exposed to permissive hyperglycemia. Zone A contained 85.92% of values; zone B contained 12.84% of values, zone D contained 1.24% of values

A total of 17 sensors were used in 15 patients. Median number of hours on CGM was 157 (IQR 59–171; range 43–214). We identified 483 paired samples (424 [87.8%] arterial blood gas values and 59 [12.2%] venous blood gas values) for assessment of CGM performance. CGM values with corresponding blood gas glucose values are shown in Figure 1 and treatment characteristics during CGM use are shown in Table S2. Overall, 13 (86.7%) CGM patients received vasopressor infusion, three (20.0%) received oral ascorbic acid, and six (40.0%) received acetylsalicylic acid. Table 4 shows CGM performance overall and across different glucose strata. Overall, CGM underestimated blood gas glucose by a median of 1.1 (95% CI, 1.0–1.3) mmol/L (mean 1.2 [95% CI, 1.1–1.4] mmol/L) or by a median of 8.4 (95% CI, 7.7–9.8)% (mean 9.0 [95% CI, 8.0–10.1]%). Median ARD and MARD were 9.6 (95% CI, 8.4–10.7)% and 11.5 (95% CI, 10.7–12.3)%, respectively. We observed 85.9% of values within Clarke zone A (CGM within 20% of blood gas glucose), 12.8% within zone B (>20% difference not leading to incorrect treatment), and 1.2% within zone D (failure to deal with hyperglycemia) (Figure 2). Bias increased with increasing blood glucose level. In contrast, we observed the lowest accuracy among blood glucose values <10 mmol/L (Table 4). Table 5 shows CGM performance in relation to sensor duration. Bias and accuracy improved with extended use beyond 48 h. Accuracy was somewhat higher when CGM was compared with venous glucose than with arterial glucose (Table S3). MARD values for individual patients ranged between 6.1% and 26.0%. We observed no clear pattern when comparing individual MARDs with the corresponding treatment charactistics during CGM use (Table S2). Reasons for CGM removal (missing data, *n* = 2) and complications (missing data, *n* = 3) associated with CGM use are presented in Table S4. One sensor was replaced due to malfunction, two were removed before surgery, and one was removed before magnetic resonance imaging. Bleeding from sensor insertion site after removal occurred in one patient.

**TABLE 4:** CGM performance calculated from the differences between CGM and blood gas glucose (CGM minus blood gas glucose)

Performance	Overall	Blood glucose range (mmol/L)		
		<10	10–14	>14
Matched pairs (*n*)	483	33	250	200
Bias				
Median difference, mmol/L	−1.1 (−1.3 to −1.0)	−0.8 (−1.5 to −0.4)	−1.0 (−1.1 to −0.8)	−1.5 (−1.8 to −1.2)
Mean difference, mmol/L	−1.2 (−1.4 to −1.1)	−0.8 (−1.2 to −0.3)	−1.0 (−1.2 to −0.9)	−1.6 (−1.8 to −1.4)
Median relative difference, %	−8.4 (−9.8 to −7.7)	−8.7 (−17.6 to −5.6)	−7.9 (−9.2 to −6.8)	−9.6 (−11.6 to −7.8)
Mean relative difference, %	−9.0 (−10.1 to −8.0)	−7.7 (−14.0 to −1.4)	−8.6 (−10 to −7.2)	−9.8 (−11.2 to −8.4)
Accuracy				
Median ARD, %	9.6 (8.4 to 10.7)	14.9 (7.0 to 20.9)	8.6 (7.8 to 10.5)	10.1 (8.5 to 11.8)
MARD, %	11.5 (10.7 to 12.3)	15.5 (11.2 to 19.7)	11.1 (10.0 to 12.2)	11.4 (10.2 to 12.5)

*Note*: Estimates are presented with 95% confidence intervals or as n (%).

Abbreviations: CGM, continuous glucose monitoring; MARD, median absolute relative difference.

**TABLE 5:** CGM performance in relation to CGM start

Performance	Hours since CGM start		
	0–48	48–96	>96
Matched pairs (*n*)	202	141	140
Bias			
Median difference, mmol/L	−1.5 (−1.7 to −1.2)	−1.0 (−1.3 to −0.9)	−1.0 (−1.2 to −0.6)
Mean difference, mmol/L	−1.4 (−1.7 to −1.2)	−1.2 (−1.4 to −1.0)	−1.1 (−1.3 to −0.9)
Median relative difference, %	−10.4 (−12.6 to −8.4)	−8.4 (−10.6 to −7.3)	−6.8 (−8.2 to −5.3)
Mean relative difference, %	−10.2 (−12.1 to −8.3)	−8.7 (−10.5 to −6.9)	−7.7 (−9.1 to −6.4)
Accuracy			
Median ARD, %	12.0 (10.3 to 14.1)	8.7 (7.7 to 10.9)	7.3 (5.8 to 8.5)
MARD, %	13.8 (12.4 to 15.1)	10.9 (9.5 to 12.3)	8.9 (7.7 to 10.0)

*Note*: Estimates are presented with 95% confidence intervals.

Abbreviations: CGM, continuous glucose monitoring; MARD, median absolute relative difference.

## 4 DISCUSSION

### 4.1 Key findings

In our before‐and‐after study of critically ill patients with type 2 diabetes exposed to liberal glucose control, we compared the performance of CGM to blood gas glucose and assessed whether CGM implementation had an impact on blood gas testing frequency and glucose variability. Compared with blood gas glucose, CGM demonstrated acceptable accuracy overall, during hyperglycemia, and up to 9 days of CGM use. Implementation of CGM reduced blood gas testing frequency but was not associated with attenuated glucose variability. No episodes of hypoglycemia developed during CGM.

### 4.2 Relationship with previous studies

Studies assessing subcutaneous CGM accuracy in ICU patients are scarce and mainly include patients with COVID‐19 monitored by a different CGM system.^9^, ^10^, ^14^ These studies report MARD values ranging from 11.1% to 13.9% and a proportion of values within Clarke zone A and B of 98%.^10^, ^14^ Our study showing an overall MARD of 11.5% and 99% of values in zone A and B support these previous findings. We observed generally lower MARD at glucose values below 10 mmol/L but only 33 paired samples were obtained in this range. Similar to the study by Faulds et al,^9^ accuracy appeared robust even in the hyperglycemia range above 14 mmol/L and during CGM use beyond 48 h. Studies assessing intravascular CGM accuracy reported slightly higher performances with MARD values between 7.7% and 9.3%^7^, ^15^ and 99.9% to 100% of values in Clarke zone A and B.^15^, ^16^ A lower performance with subcutaneous CGM could partly be explained by the expected lag‐time between the blood and interstitial compartments dictated by the glucose diffusion rate from blood and interstitium as well as the speed of cellular glucose uptake.^17^ Poor tissue perfusion, commonly seen in critically patients, may further enhance this lag‐time and impair accuracy.

In previous studies, CGM use was associated with a 60%–70% reduction in the number of routine blood glucose tests.^9^, ^10^, ^11^ In contrast, we observed a reduced sampling frequency of approximately 30%. The lower reduction observed by us is expected. Blood gas analysis of glucose was standard care in our unit whereas previous studies used POC meters. Since blood gases are frequently obtained for assessment of additional biomarkers (e.g., lactate and partial pressure of oxygen), they will not be completely replaced by CGM. Moreover, we compared our findings with the actual sampling frequency in a pre‐CGM control group, whereas previous studies compared with an assumed hourly sampling frequency.

Time in target glucose range was low in our study (50% in the CGM group and 48% in the No CGM group). Similar results were observed in the intervention group (45.5% of values in target range) of a recent multicenter trial comparing liberal with conventional glucose control in the ICU.^18^

### 4.3 Study implications

Our study findings imply that CGM is a feasible and clinically acceptably accurate modality in critically ill patients with type 2 diabetes exposed to liberal glycemic control for up to 9 days in ICU. Our results further imply that CGM implementation may reduce blood gas sampling frequency and bedside nurses' workload. However, we observed no robust association with glucose variability; CGM was associated with reduced coefficient of variation but not with a reduction in standard deviation or glycemic lability index. The lower coefficient of variation among CGM patients is most likely explained by a higher mean glucose level in this group. With no CGM readings in the hypoglycemic range, we were unable to assess whether CGM use has the potential to prevent hypoglycemia.

### 4.4 Strengths and limitations

Our study has several strengths. It is the first study to assess CGM performance in critically ill patients exposed to liberal glucose control. In addition, we used blood gas glucose as reference method, which has similar accuracy as laboratory measurements.^19^ This is important as glucose measurement by POC meters are considered less reliable in the ICU setting.^20^, ^21^ Finally, we compared several glucose metrics with a control population of critically ill patients with type 2 diabetes treated according to an identical glucose management protocol.

We acknowledge several limitations. Our study is not a randomized controlled trial. Therefore, residual confounding cannot be ruled out. However, our pragmatic approach is a necessary step to inform the design of future randomized efficacy trials. Sample size was relatively small with insufficient power to assess the potential impact of patient‐specific, illness severity‐related and treatment‐related factors on CGM performance. The observed variation of MARD values between patients was not clearly explained by differences in the treatment characteristics described in this study (renal replacement therapy, vasopressors, milrinone, ascorbid acid and acetylsalicylic acid). Assessment of factors potentially interfering with CGM performance will be an important next step, but requires a larger cohort. Liberal glucose control is not universally applied to ICU patients with type 2 diabetes. Since our data mainly consisted of blood glucose values in the hyperglycemic range, generalizability of our findings to ICUs using stricter glucose control protocols should be done with caution. Less accuracy during the first 48 h may also limit utility during this time‐frame.

## 5 CONCLUSIONS

In our cohort of ICU patients with type 2 diabetes managed according to a liberal glucose control protocol, CGM showed acceptable accuracy and was associated with a significant reduction in blood gas sampling frequency without compromising blood glucose control. However, CGM was less accurate at glucose values below 10 mmol/L and during the first 48 h of CGM use. Our findings justify further assessment of the value of CGM in preventing hypoglycemia and attenuating glucose variability in the ICU and whether such potential benefits translate into improved clinical outcomes.

## AUTHOR CONTRIBUTIONS

Concept was done by Rinaldo Bellomo, Salvatore Cutuli, Fumitaka Yanase, and Johan Mårtensson. Patient recruitment and data collection was done by Salvatore Cutuli, Fumitaka Yanase, Paolo Ancona, Lisa Toh, and Eduardo Osawa. Johan Mårtensson conducted the statistical analyses. Johan Mårtensson and Rinaldo Bellomo wrote the first draft. Editing of the manuscript was done by all authors. All authors read and approved the final manuscript. The manufacturer of the FreeStyle Libre (Abbott) was not involved in the study design, study execution or interpretation or reporting of study results.

## FUNDING INFORMATION

Johan Mårtensson received financial support through the regional agreement on medical and clinical research (ALF) between Stockholm County Council and Karolinska Institutet. The study did not receive any financial support from Abbott. Abbott was not involved in the study design, study execution or interpretation or reporting of study results.

## CONFLICT OF INTEREST

The authors report no conflicts of interest.

## Supporting information


**Appendix S1** Supporting Information
