# Supplementary material for: Glycemic control and blood gas sampling frequency during continuous glucose monitoring in the intensive care unit: A before‐and‐after study
Source: Acta Anaesthesiol Scand. 2022 Oct 28;67(1):86–93. doi: 10.1111/aas.14159 (PMC10092568; doi:10.1111/aas.14159)
Supplement: Supplementary file 1 — Appendix S1 Supporting Information [file AAS-67-86-s001.docx]

**Supplementary Material**

**Table S1**. Univariable and multivariable linear regression analysis showing the association between CGM use and number of blood gas glucose values and glucose variability (standard deviation, coefficient of variation and glycemic lability index), respectively

| **Outcome measure** | **Unadjusted risk estimate (95% CI)** | **P value** | **Adjusted risk estimate^a^ (95% CI)** | **P**  **value** |
| --- | --- | --- | --- | --- |
| Number of blood gas glucose measurements per hour | -0.11 (-0.17 to -0.06) | <0.001 | -0.12 (-0.18 to -0.07) | <0.001 |
| Standard deviation of blood gas glucose, mmol/l | 0.87 (0.72 to 1.05) | 0.15 | 0.90 (0.74 to 1.08) | 0.24 |
| Coefficient of variation of blood gas glucose, % | 0.77 (0.65 to 0.90) | 0.002 | 0.78 (0.66 to 0.92) | 0.004 |
| Glycemic lability index of blood gas glucose, [mmol/l]^2^/h/week | 0.95 (0.60 to 1.50) | 0.82 | 1.02 (0.64 to 1.65) | 0.92 |
| ^a^Adjusted for APACHE III score and ICU length of stay | | | | |

**Table S2**. Treatment characteristics during CGM use and sensor accuracy (MARD)

| **Patient**  **#** | **RRT** | **Maximum**  **noradrenaline**  **infusion rate**  **(µg/min)** | **Maximum**  **Vasopressin**  **infusion rate**  **(units/min)** | **Maximum**  **Milrinone**  **infusion rate**  **(µg/kg/min)** | **Ascorbic acid**  **(g/d)** | **Acetyl-salicylic acid**  **(mg/d)** | **MARD**  **(%)** |
| --- | --- | --- | --- | --- | --- | --- | --- |
| 1 | No | 0 | 0 | 0 | 0 | 0 | 23.5 |
| 2 | No | 0 | 0 | 0 | 0 | 0 | 8.5 |
| 3 | No | 8 | 0 | 0 | 0 | 0 | 6.1 |
| 4 | Yes | 13 | 0 | 0 | 0.5 (oral) | 100 | 15.2 |
| 5 | No | 4 | 0 | 0 | 0 | 100 | 10.7 |
| 6 | No | 4 | 0 | 0.06 | 0 | 100 | 12.6 |
| 7 | No | 8 | 0 | 0 | 0 | 100 | 8.6 |
| 8 | Yes | 6 | 0 | 0 | 0.5 (oral) | 0 | 8.2 |
| 9 | No | 7 | 0 | 0 | 0 | 0 | 7.6 |
| 10 | Yes | 24 | 0 | 0.1 | 0.5 (oral) | 0 | 15.6 |
| 11 | No | 0.5 | 0 | 0 | 0 | 0 | 10.1 |
| 12 | No | 3 | 0 | 0 | 0 | 0 | 12.0 |
| 13 | No | 10 | 0 | 0 | 0 | 100 | 11.9 |
| 14 | No | 6 | 0 | 0 | 0 | 0 | 13.8 |
| 15 | No | 12 | 0 | 0.25 | 0 | 100 | 26.0 |
| RRT = renal replacement therapy, MARD = mean absolute relative difference between CGM and blood gas glucose | | | | | | | |

**Table S3**. CGM performance calculated from the differences between CGM and blood gas glucose (CGM minus blood gas glucose)

| **Performance** | **CGM versus arterial blood glucose** | **CGM versus venous blood glucose** |
| --- | --- | --- |
| Matched pairs (n) | 424 | 59 |
| Bias |  |  |
| Median difference, mmol/l | -1.2 (-1.4 to -1.0) | -0.9 (-1.3 to -0.4) |
| Mean difference, mmol/l | -1.3 (-1.4 to -1.1) | -1.0 (-1.4 to -0.6) |
| Median relative difference, % | -9.1 (-10.3 to -7.9) | -6.3 (-8.4 to -4.3) |
| Mean relative difference, % | -9.3 (-10.4 to -8.2) | -7.3 (-10.1 to -4.5) |
| Accuracy |  |  |
| Median ARD, % | 10.1 (9.0 to 11.3) | 6.8 (5.5 to 9.4) |
| MARD, % | 11.7 (10.9 to 12.6) | 9.8 (7.6 to 12.0) |
| Estimates are presented with 95% confidence intervals | | |

**Table S4**. Reasons for CGM sensor removal and complications

| **Variable** | **All CGM sensors**  **(n = 17)** |
| --- | --- |
| Reasons for sensor removal |  |
| Malfunction | 1 (5.9) |
| Surgery | 2 (11.8) |
| Magnetic resonance imaging | 1 (5.9) |
| End of planned session | 7 (41.2) |
| Discharged from the intensive care unit | 4 (23.5) |
| Missing data | 2 (11.8) |
| Complications |  |
| No complications | 13 (76.5) |
| Bleeding | 1 (5.9) |
| Missing data | 3 (17.6) |
| Data are n (%) | |


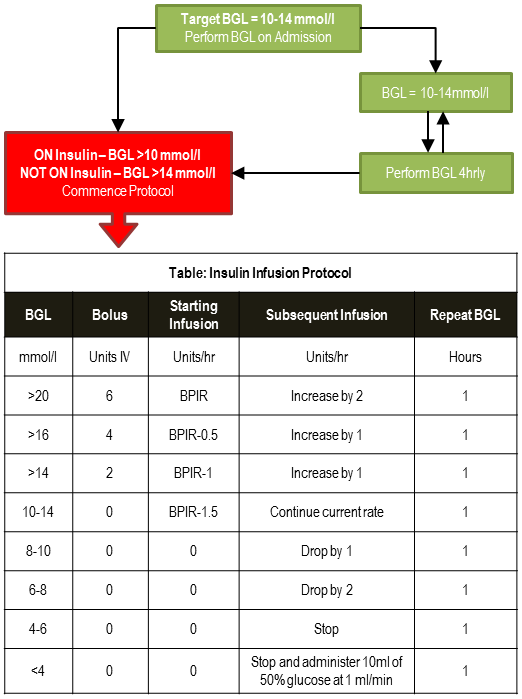


**Figure S1.** Liberal protocol for blood glucose management of patients with diabetes at the Austin Hospital Intensive Care Unit since 2015. Baseline pre-morbid insulin requirements **(BPIR)** is for diabetic patients who are previously receiving insulin and is the total insulin that the patient is receiving prior to their acute illness divided by 24 (hours).
